# Comprehensive encoding of conformational and compositional protein structural ensembles through the mmCIF data structure

**DOI:** 10.1107/S2052252524005098

**Published:** 2024-06-25

**Authors:** Stephanie A. Wankowicz, James S. Fraser

**Affiliations:** a Department of Bioengineering and Therapeutic Science University of California San Francisco CA94117 USA; Chinese Academy of Sciences, China

**Keywords:** biomolecules, macromolecular ensembles, ensemble–function predictions, mmCIF, cryoEM

## Abstract

In the folded state, biomolecules exchange between multiple conformational states crucial for their function. However, most structural models derived from experiments and computational predictions only encode a single state. To represent biomolecules accurately, we must move towards modeling and predicting structural ensembles. Information about structural ensembles exists within experimental data from X-ray crystallography and cryo-electron microscopy. Although new tools are available to detect conformational and compositional heterogeneity within these ensembles, the legacy PDB data structure does not robustly encapsulate this complexity. We propose modifications to the macromolecular crystallographic information file (mmCIF) to improve the representation and interrelation of conformational and compositional heterogeneity. These modifications will enable the capture of macromolecular ensembles in a human and machine-interpretable way, potentially catalyzing breakthroughs for ensemble–function predictions, analogous to the achievements of *AlphaFold* with single-structure prediction.

## Abstract 2

Traditional structural models of biomolecules typically represent only a single conformational state, even though biomolecules naturally exist in multiple states crucial for their function. Here, we propose enhancements to the macromolecular crystallographic information file (mmCIF) to better capture the complex conformational and compositional heterogeneity of biomolecules that is human- and machine-interpretable.

## 1. Introduction

Most structural models deposited in the Protein Data Bank (PDB) (Burley, Berman *et al.*, 2017 ▸) result from experimental X-ray crystallography or single-particle cryoEM studies. These methods collect data averaged over tens of thousands to billions of individual copies of the system (containing macromolecules, solvent, ions, small molecules *etc.*). Each molecule within the system can adopt a different conformation (conformational heterogeneity) and may differ slightly in chemical composition (compositional heterogeneity). However, structural models generally represent the system with a single coordinate set. This simplification overlooks the multiple states present in the experimental data and consequently omits details vital to understanding protein function (Furnham *et al.*, 2006 ▸; Holton *et al.*, 2014 ▸). The single coordinate set convention originated in the specifications of the legacy PDB format and is perpetuated in the current PDBx/mmCIF standard (Westbrook *et al.*, 2022 ▸). However, with the universal adoption of PDBx/mmCIF (Adams *et al.*, 2019 ▸), we propose to expand our representations to separate these two aspects of heterogeneity within the sample, enabling more precise and accurate structures to train deep-learning approaches that model dynamic biomolecular systems.

**Figure 1:**
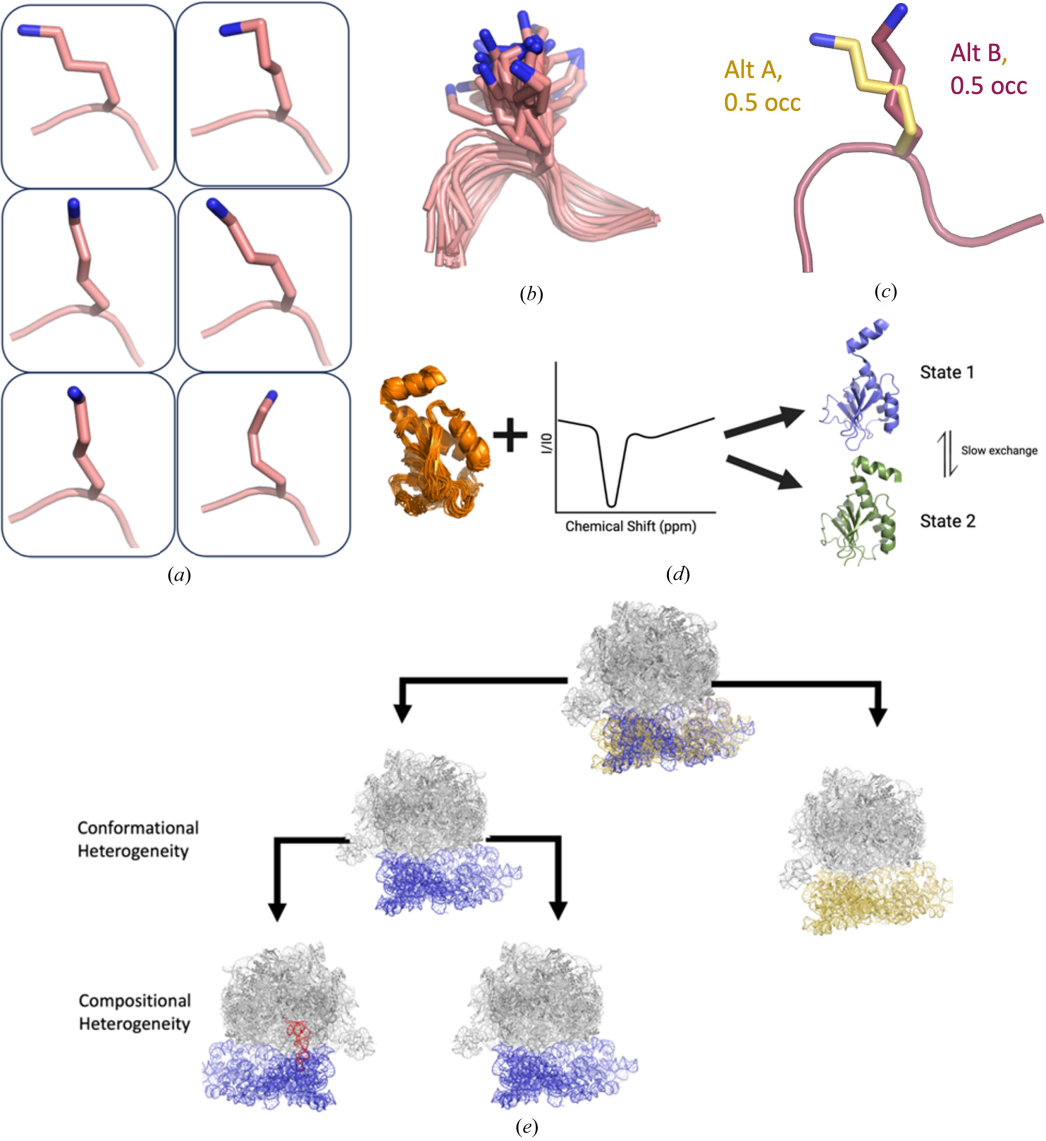
Model types to represent conformational heterogeneity. (*a*) Examples of the multiple conformations of protein side chains captured by cryoEM and X-ray crystallography. (*b*) Ensemble representation of a Lys side chain. (*c*) Multiconformer representation of a Lys side chain. (*d*) One-state NMR ensemble from distant restraints and a two-state NMR ensemble from CEST data. (*e*) 3D classification from cyroEM results in different but related maps, often with distinct associated models.

In cases where the deposited models go beyond a single set of coordinates, capturing the underlying experimental ensemble is solved in two primary ways: ensemble models that encode many copies of the system, each with a potentially distinct conformation/composition; in a single PDB deposition or multiconformer models that encode alternate conformational/compositional states only for certain parts of the system (Fig. 1 ▸). Nuclear magnetic resonance (NMR) data are typically encoded in ensemble models because they inferentially represent sparse distance and angle restraints (Rieping *et al.*, 2005 ▸). Occasionally, these conventional restraints are augmented with other NMR observables, such as relaxation dispersion measurements, that more directly report on distinct states. There are proposals for the best ways to represent the multiple ensembles necessitated by their inclusion (Ramelot *et al.*, 2023 ▸). In contrast, cryoEM and X-ray crystallography density maps provide atomistic detail across the entire system, enabling precise modeling of alternate states directly from a real space signal. As there is currently no principled way of choosing the most parsimonious ensemble size, encoding cryoEM and X-ray crystallography data in ensemble models can result in an exploding data-to-parameter ratio and overfitting [Fig. 1 ▸(*b*)] (Burnley *et al.*, 2012 ▸; Babcock *et al.*, 2018 ▸). Moreover, ensemble models are difficult to modify manually [*e.g.* in *Coot* or *ISOLDE* (Emsley *et al.*, 2010 ▸; Croll, 2018 ▸)]. In contrast, multiconformer models can represent all states within a single model, reducing the data-to-parameter ratio and allowing for more facile human visualization and manipulation (Wankowicz *et al.*, 2023 ▸; Riley *et al.*, 2021 ▸; Stachowski & Fischer, 2023 ▸). A limitation of the current PDBx/mmCIF data structure for ensemble and multiconformer models is that it cannot represent the complex interdependencies of alternative conformational states in the experimental ensemble.

Modeling the experimental ensemble also requires representing the chemical compositional heterogeneity. This heterogeneity can result from covalent modification (*e.g.* post-translational modification) or the presence of a binding partner stabilized by non-covalent interactions (*e.g.* a subunit of a macromolecular complex, a small-molecule ligand or even a solvent molecule). Using ensemble-based approaches leads to the same model size selection problems outlined above. Refining the weight of different conformational ensemble members in the modified/bound state also connects this heterogeneity more naturally to the multiconformer format. A major issue for encoding compositional heterogeneity in multiconformer models is that the current formats use the exact same representation for conformational and compositional heterogeneity, creating ambiguity about the various states present in the models and their relationship to the experimental ensemble.

Here, we propose amendments to the PDBx/mmCIF model format (Westbrook *et al.*, 2022 ▸) to improve the encoding of the conformational and compositional ensembles in experimental structural biology data. Using the extensible and flexible dictionary based data structure of the mmCIF/PDBx format, we propose separated entities to capture conformational and compositional heterogeneity that can be layered to show hierarchical relationships (Fig. 1 ▸). These modifications will improve our ability to explain structural ensembles and provide critical training data for new protein ensemble–function predictions.

## 2. The current PDBx/mmCIF format inadequately captures conformational and compositional heterogeneity

The failure of deposited structures in the Protein Data Bank (PDB) to represent the underlying experimental conformational and compositional heterogeneity is partly attributable to the complexity of modeling in the presence of limited signal-to-noise (Lane, 2023 ▸). Noise arises from many sources, including crystal imperfections and radiation damage in X-ray crystallography (Weichenberger *et al.*, 2015 ▸; Karplus & Diederichs, 2012 ▸), and beam-induced motion and imperfect detector quantum efficiency (DQE) in cryo-EM (Glaeser, 2019 ▸). Additionally, poor modeling resulting from inaccurate phases (for X-rays) and errors in particle alignment and classification (for cryoEM) dominate the imperfect agreement between experiment and model. Further complicating the discovery of heterogeneity is that conformational heterogeneity manifests in many forms. A high amount of harmonic heterogeneity manifests in a fall off of density from a mean atomic position. This type of heterogeneity can be modeled in the PDB format by isotropic, anisotropic or grouped [*e.g.* translation–libration–screw (Winn *et al.*, 2001 ▸; Afonine *et al.*, 2018 ▸)] *B* factors that fit the extent of the disorder (Konnert & Hendrickson, 1980 ▸; O’Connor, 1975 ▸).

Additionally, many macromolecular motions have a highly anharmonic character (*e.g.* rotamer jumps or sub-domain opening) that manifests in discrete but weaker density around distinct positions with no continuous density connecting the states. This type of heterogeneity is not well fit by *B* factors, which leads to underestimation of the displacements present in the experimental ensemble (Kuzmanic *et al.*, 2014 ▸; Kuriyan *et al.*, 1986 ▸). To overcome this limitation in PDB format, atoms can be replicated and labeled with an ‘alternative location indicator’ (altloc), signifying discrete states. Refinement and validation programs treat atoms sharing the same altloc as having the ability to interact with each other and with atoms lacking an altloc, but not with atoms with different altlocs. However, the lack of a hierarchical relationship between altlocs restricts the complexity of information encoded by the legacy format.

Capturing ensemble information in the legacy PDB format becomes an even more complex problem when considering compositional heterogeneity, which can coexist with conformational heterogeneity. Compositional heterogeneity is often observed with ligands bound at sub-stoichiometric occupancy in X-ray structures (Danley, 2006 ▸; Turnbull & Emsley, 2013 ▸; Müller, 2017 ▸) and with different components in large macromolecular complexes in cryoEM (Webster *et al.*, 2023 ▸). Compositional heterogeneity is captured using the same ‘altloc’ column as conformational heterogeneity. This ambiguous representation inhibits disentangling compositional and conformational heterogeneity, especially for large data-mining efforts.

Computational tools have recently improved in decoding the complex conformational and compositional heterogeneity signal from the noise. In cryoEM, human intervention or machine-learning tools can distinguish different large conformations. While many of these tools are primarily used for visualization, some can incorporate discrete states into heterogeneous refinement, moving towards ensemble-based cryoEM models (Zhong, Bepler *et al.*, 2021 ▸; Punjani & Fleet, 2021 ▸; Serna, 2019 ▸). In X-ray crystallography and cryoEM, methods exist that automatically detect subtle conformational shifts, like rotamer jumps, among structural ensemble members through multiconformer approaches (Wankowicz *et al.*, 2023 ▸; Keedy *et al.*, 2015 ▸; Riley *et al.*, 2021 ▸; Stachowski & Fischer, 2023 ▸; Ginn, 2021 ▸). Further, weak signals representing compositional heterogeneity, often observed in X-ray ligand-soaking experiments, can now be more easily identified using approaches such as *PanDDA* (Pearce *et al.*, 2017 ▸). In cryoEM, compositional heterogeneity often occurs by having subsets of complex subunits on the grid throughout the data collection. This can been studied by exploring differences in the same or related maps (Punjani & Fleet, 2023 ▸; Rabuck-Gibbons *et al.*, 2022 ▸; Zhong, Bepler *et al.*, 2021 ▸; Powell & Davis, 2024 ▸).

However, these tools are confined by the data structure that must represent their output in the Protein Data Bank. Failing to account for the diverse conformational and compositional states hinders a thorough understanding of biological functions, the precision of predictive modeling, and the innovation in designing novel proteins and small-molecule inhibitors.

## 3. Alterations to the existing mmCIF format can capture conformational and compositional heterogeneity in structures

The PDBx/mmCIF is an extensible data representation built on a flexible dictionary based system (Bourne *et al.*, 1997 ▸; Westbrook *et al.*, 2022 ▸). While this data format allows for a more robust representation of many structural models, conformational and compositional heterogeneity is encoded in the same way as the legacy PDB format (altlocs and *B* factors). However, conformational and compositional heterogeneity represent two very different types of heterogeneity. Conformational heterogeneity refers to different conformations of the same species (*i.e.* different atomic coordinates) within the same dataset, whereas compositional heterogeneity refers to different species (*i.e.* small molecules or monomers of a macromolecular complex) within the same dataset.

We propose extending the PDBx/mmCIF model to include new conformational and compositional data categories linked to atom-level data (Fig. 1 ▸). Additionally, both data categories would be hierarchical. For each atom, the first conformational state would represent the base or first layer of heterogeneity, with subsequent states explaining heterogeneity ‘within’ the previous state. For example, in the conformational data category, conformational state level 1 could contain information on a loop state (in or out), whereas conformational state level 2 would contain a backbone peptide flip in the out loop state, and conformational state 3 could contain information about a side chain alternative conformation that occurs in a residue in the out loop state and with the peptide flip. This layering would allow us to build our knowledge of hierarchical conformational heterogeneity. Isotropic and anisotropic atomic displacement parameters (*B* factors) would still exist in the atom-level data across the hierarchy. It would be straightforward to continue to refine TLS-derived atomic displacement parameters for non-multiconformer regions; however, the exact implementation of how TLS-derived groupings will co-exist with hierarchical representations of conformational heterogeneity will require careful attention in refinement software. Separating compositional and conformational heterogeneity into individual data categories allows us to understand how they are linked, such as multiple conformations populated in the liganded state. Importantly, the inherent flexibility of the mmCIF format (Westbrook *et al.*, 2022 ▸) paves the way for a standardized and adaptable format to capture conformational and compositional heterogeneity depending on the experimental data.

In the following sections, we present various examples that contrast the current representation in the mmCIF format (most of which are holdovers from the legacy PDB format) versus our envisioned depiction. We discuss how these changes can be integrated with refinement protocols.

## 4. Example 1: simple conformational heterogeneity

**Figure 2:**
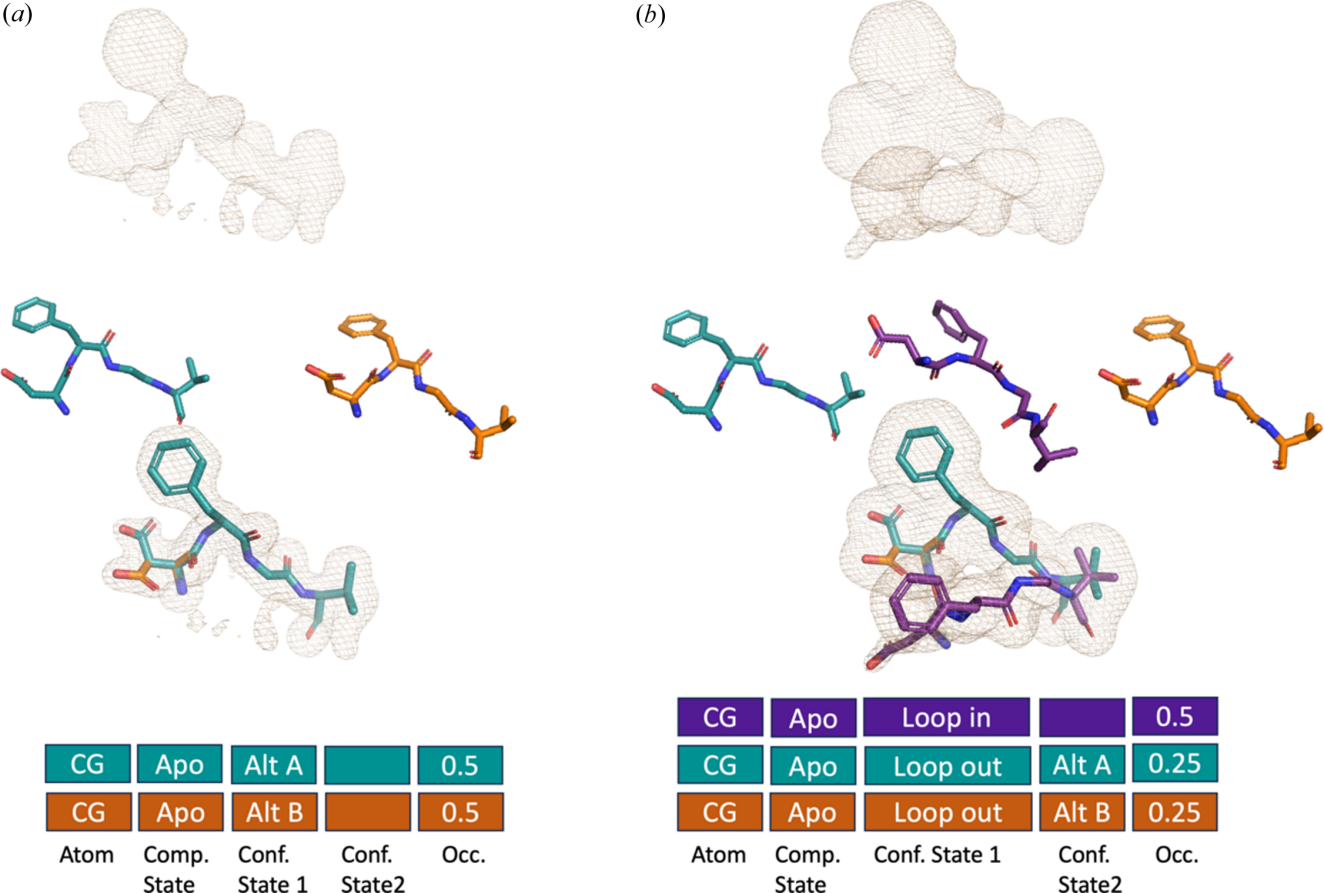
Conformational heterogeneity. Boxes represent how data categories would be connected in the mmCIF format. We are using the CG atom on the Asp residue as our example. The categories shown are: atom (CG), compositional state, conformational state 1, conformational state 2 and occupancy. (*a*) Example 1: simple conformational heterogeneity with a single residue. (*b*) Example 2: layer conformational heterogeneity with layer one conformational heterogeneity being a loop and layer two conformational heterogeneity being additional heterogeneity in the Asp residue.

The simplest example is an apo protein with alternative conformations of single residues or sections of residues. Currently, these could be captured by alternative conformations or increased *B* factors. In our proposed mmCIF format, the first conformational data category would represent the alternative positions of an individual residue [Fig. 2 ▸(*a*)] or multiple residues, such as a loop [Fig. 2 ▸(*b*)]. We envision this ‘conformational state 1’ category to be within the atom_site loop of the mmCIF dictionary and optional. We would allow users to use any REGEX categories to identify each conformation and have an additional data category with the description for each ID to inform others of what each state means. In this scenario, refinement software would work exactly as it is now by constraining the occupancy of each atom to sum to one, but the altloc names will be longer than a single character. We also propose that ‘altloc’ in the current mmCIF format be moved into the conformational state 1 data category. Further, existing ensemble structures could be trivially converted to this data format by having each model in the ensemble have a different identifier in the conformational state 1 data category.

## 5. Example 2: layered conformational heterogeneity

Next, a more complicated scenario with the apo protein to demonstrate hierarchically related conformational heterogeneity, such as an alternative conformation within a loop. For example, in the ‘loop out’ conformation (state 1), there are multiple positions of a single leucine side chain (state 2) [Fig. 2 ▸(*b*)]. In the current mmCIF format, you could encode the three conformations as A (loop in), B (loop out, position 1) and C (loop out, position 2), but these would have no descriptive or hierarchical relationship to each other. In our proposed mmCIF additions, we could encode the loop conformational heterogeneity in conformational state 1 and the leucine conformational heterogeneity within conformational state 2. We see this as another category of conformational heterogeneity state 2 within the atom_site loop, and it has the exact requirements of the other conformational heterogeneity layer. However, to have a conformational heterogeneity state 2 category, the atom must have a conformational heterogeneity state 1 category. For refinement, restraints would be linked to each level of conformation, such that all conformations in conformational heterogeneity state 1 (loop in and loop out) would have to equal 1, while the categories in conformational heterogeneity state 2 (conformations in loop out) would have to sum to occupancy of the conformational heterogeneity state 1 class it belongs in (loop in) [Fig. 2 ▸(*b*)]. Clashes could be evaluated in the PDB deposition validation by all atoms with the same label at the same hierarchy level, extending the current validation scheme (Read *et al.*, 2011 ▸). A full example of PDB entry 6b90 (Keedy *et al.*, 2018 ▸) represented in the historical PDB format, proposed mmCIF and ensemble representation is available in the supporting information.

## 6. Example 3: simple compositional heterogeneity

**Figure 3:**
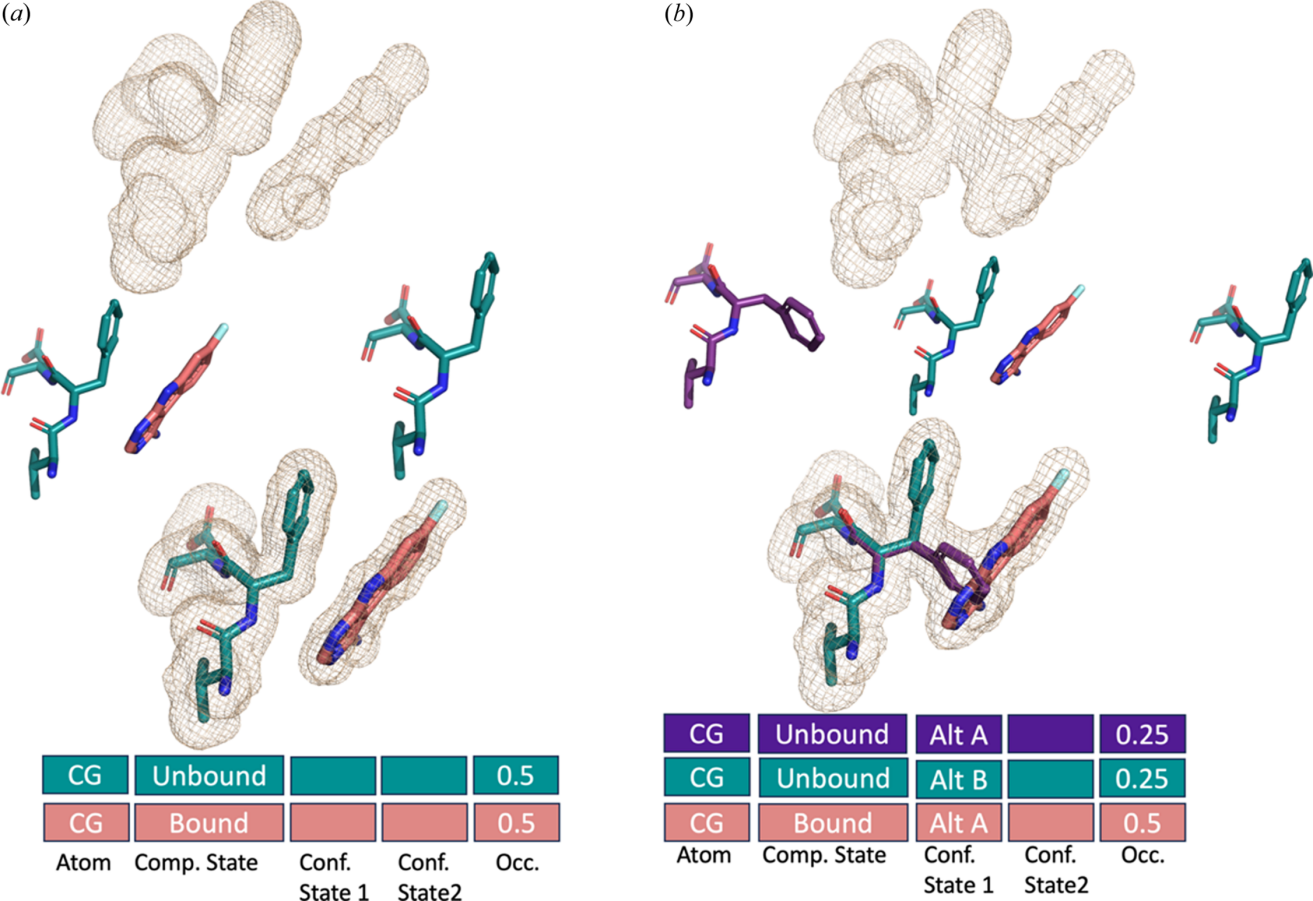
Compositional heterogeneity. Boxes represent how data categories would be connected in the mmCIF format. Shown are: atom, compositional state, conformational state 1, conformational state 2, occupancy. Information from the CG atom of the Phe residue is shown. (*a*) Example 3: simple compositional heterogeneity, where the ligand has 50% occupancy (bound state). (*b*) Example 4: compositional and conformational heterogeneity, where the ligand has 50% occupancy (bound state), and the Phe exists in two conformations in the other 50% (or unbound state)

Next, we have a protein with a partially occupied ligand (*i.e.* present in 50% of the protein copies) in a space that does not clash with the apo state of the protein. Compositional heterogeneity is almost always observed in high-throughput ligand soaking experiments, which now make up a huge percentage of the PDB depositions (Gahbauer *et al.*, 2023 ▸; Barthel *et al.*, 2022 ▸; Skaist Mehlman *et al.*, 2023 ▸), and is increasing in frequency in deposited cryoEM structures. However, representing these data has been a topic of great debate (Weiss *et al.*, 2022 ▸; Jaskolski *et al.*, 2022 ▸). In the legacy PDB format, we could encode the compositional heterogeneity by indicating that the small molecule has an occupancy of 0.5 (50%) and provide it with an altloc, often conflated with conformational heterogeneity [Fig. 3 ▸(*a*)]. In the proposed format, we proposed separating compositional heterogeneity from conformational heterogeneity. We propose a compositional state data category that would also be within the atom_site loop and have a dictionary of categories based on the combination of each unique, non-water HETATM (*i.e.* ligand 1 bound, ligand 1 and 2 bound, ligand 2 bound, unbound, or blank indicating unknown and therefore interacting with all compositional states). For every atom, the user can choose if this is bound, unbound or unknown for each HETATM. For the ligands themselves, they will always be in the bound state, but for the other atoms around they can be in the bound, unbound or unknown state. The unknown state will consist of most of the atoms in the protein as it is typically unknown if the atom is in the unbound or bound state, especially in residues far from the binding site. This also allows for ligand 1 bound to be indicated in the compositional heterogeneity category and pose 1 or 2 in the conformational heterogeneity column.

## 7. Example 4: compositional and conformational heterogeneity

We now consider building on this example of a partially occupied ligand with conformational heterogeneity in the protein. The interplay between conformational and compositional heterogeneity is often inferred from the ligand clashing with some protein conformations [Fig. 3 ▸(*b*)]. In this example, the ligand binding is compatible with the ‘loop in’, but not the ‘loop out’ conformation. In addition, there are individual residues in each loop state. The complexity of this interlinked conformational and compositional heterogeneity would be completely lost in the legacy PDB format (see the supporting information). We would have to encode the conformational heterogeneity of the protein with at least four altloc IDs and make copies of the ligand that match the altloc IDs of the compatible conformations. There would be no link between how the conformational heterogeneity interacts with the compositional heterogeneity. Furthermore, the hierarchy of conformational heterogeneity is also lost. This encoding is critical in certain time-resolved experiments, where proteins are perturbed with temperature (Thompson *et al.*, 2019 ▸), electric field (Greisman *et al.*, 2023 ▸) or light (De Zitter *et al.*, 2022 ▸). In these experiments, the ground and excited states can contain the same conformational states, although at different occupancies. With hierarchy and different compositional states, it would be possible to represent both states within the same PDB entry.

In our proposed mmCIF model, within the atom_site, we would encode compositional heterogeneity (bound or unbound or unknown for ligand 1) and conformational heterogeneity state 1/2. For example, when the compositional column indicated a ‘bound’ state, the corresponding conformational state 1 would indicate the alt A. However, when the ligand was not bound, the compositional state will be unbound but the conformational state 1 could indicate alt A or alt B. The occupancies of the conformations in the bound state would be restrained to the sum of the bound occupancy. For residues that do not interact with the ligand, we would imagine that the compositional column would be blank or unknown (see the supporting information). This concept can be extended to subunits in assemblies from cryoEM data or covalent linkages, such as a post-translational modification, as presented in Fig. 1 ▸(*e*).

## 8. Conclusions

The development of deep-learning methods to predict the single-structure representations in the PDB have been a breakthrough for structural biology (Jumper *et al.*, 2021 ▸; Baek *et al.*, 2021 ▸). However, the next challenge lies in predicting ensembles. This is important for two reasons: first, ensembles dictate function; and second, the accuracy gap between prediction methods and experiments may result from an incomplete consideration of ensembles on both sides (Lane, 2023 ▸). A substantial upgrade in representing our experimental structural data is needed to meet this challenge.

X-ray crystallography and single-particle cryoEM capture an ensemble of atomistic information, enabling the modeling of compositional and conformational states. Numerous methods exist to disentangle these states (Wankowicz *et al.*, 2023 ▸; Riley *et al.*, 2021 ▸; Keedy *et al.*, 2015 ▸; Ginn, 2021 ▸; Ploscariu *et al.*, 2021 ▸; Pearce *et al.*, 2017 ▸; Hoff *et al.*, 2023 ▸), but the current PDBx/mmCIF format inadequately captures these states. We also believe this proposal can be expanded to other structural biology techniques such as neutron crystallography (Catapano *et al.*, 2023 ▸). While it is possible to capture these data with ensemble data representation, there can be an explosion of the number of states due to the atomistic detail. Ensemble representations in NMR result from inferential determination of positions based on highly local observables, which face this issue and generally represent a fixed number without any Boltzmann weighting implied (Ramelot *et al.*, 2023 ▸). Similarly, recent integrative modeling approaches that rely heavily on distance restraint and other non-atomistic data can be deposited in the PDB-Dev (Burley, Kurisu *et al.*, 2017 ▸; Vallat *et al.*, 2019 ▸). However, these integrative and inferential modeling challenges are distinct from those faced by real and reciprocal space modeling in X-ray crystallography and cryoEM in an important way: integrative approaches are generally underdetermined and inferential, whereas X-ray/EM density maps are generally overdetermined for a single model.

Our proposed amendments to the PDBx/mmCIF model are designed to better represent conformational and compositional heterogeneity by capturing these heterogeneities with new data categories in PDBx/mmCIF (see the supporting information). This modification will separate out conformational and compositional heterogeneity and allow for information to be transferred about the interconnectedness of this heterogeneity. We propose adding these data categories onto the atom_site loop within the PDB, connecting each atom in a structure to its conformational or compositional state. While a data category in the PDBx/mmCIF dictionary allows for more description of conformational heterogeneity (alt_ens), this does not separate conformational versus compositional heterogeneity, nor does it allow for layering of conformational heterogeneity.

This improved data structure will accelerate the development of new tools and create representative training datasets for structural ensemble prediction. Alongside this format, infrastructure changes to refinement, visualization and validation tools are likely to be needed. We have outlined changes here to maximize backward compatibility for refinement and visualization software; for example, the hierarchical representations can be implemented, at first, as parsable group occupancy definitions. This new format should also help with the interconversion of existing multiconformer and ensemble-based models. Such interconversion enables manual manipulations, such as in *Coot* (Emsley *et al.*, 2010 ▸) or data mining approaches. We envision refining mmCIF further to more effectively correlate grouped data, including time-resolved techniques, ligand soaking experiments or EM classification/reconstructions (Zhong, Lerer *et al.*, 2021 ▸; Zhong, Bepler *et al.*, 2021 ▸). For example, a ‘perturbation’ data category could connect to specific structure factors or real-space maps, enabling restrained refinements of coordinates.

The notion that a single, static structure defines a protein is outdated for experimental and structural prediction. Macromolecules adopt an ensemble of conformations, and modeling those structural distributions accurately is now possible. By more correctly encapsulating the underlying experimental data, we can enable both benchmarks for prediction and a new class of ensemble–function studies. Moreover, accurately modeling compositional heterogeneity will reveal how ligands interact with the receptors, increasing the potential for an ‘*AlphaFold*’-type of breakthrough in ligand design. Inevitably, all models are wrong, but we can at least aspire to make more useful models that take advantage of the expressive mmCIF format to better model the heterogeneity in the underlying experimental data.

## Supplementary Material

Historic PDB representation. DOI: 10.1107/S2052252524005098/lz5069sup1.txt

Proposed mmCIF. DOI: 10.1107/S2052252524005098/lz5069sup2.txt
